# Tacrolimus trough levels in kidney transplant recipients

**DOI:** 10.1186/s12882-021-02622-5

**Published:** 2021-12-07

**Authors:** Young Hui Hwang, Hyunjung Kim, Kyungok Min, Jaeseok Yang

**Affiliations:** 1 Department of Nursing, College of Medicine, University of Ulsan, Ulsan, South Korea; 2 Divison of Nursing & Research Institute of Nursing Science, Hallym University, 1 Hallymdaehak-gil, Chuncheon, Gangwon-do 24252 South Korea; 3 Transplant Center, Department of Nursing, Seoul National University Hospital, Seoul, South Korea; 4 Division of Nephrology, Department of Internal Medicine, Yonsei University College of Medicine, Seoul, South Korea

**Keywords:** Coefficient of variation, Mean, Medication adherence, Standard deviation, Tacrolimus trough level

## Abstract

**Background:**

It is very important that kidney transplant recipients (KTRs) take immunosuppressive drugs to prevent graft rejection. This study aimed to identify the tacrolimus trough levels (TTL)-mean, TTL-standard deviation (SD), and TTL- coefficient of variation (CV) as well as factors affecting these values over a 2-year period in clinically stable patients > 5 years after kidney transplantation (KT).

**Methods:**

This retrospective study analyzed data from 248 adult outpatients > 5 years after KT. Medical chart data, including TTL, graft rejection, and tacrolimus dose change during a 2-year period, between January 2017 and December 2018, were collected. Multivariable regression analyses were conducted to determine the factors influencing the TTL-mean, TTL-SD, and TTL-CV.

**Results:**

The TTL-mean, TTL-SD, and TTL-CV were 6.00 ± 1.07 ng/mL, 1.51 ± 1.09 ng/mL, and 0.25 ± 0.14, respectively. The TTL-mean, TTL-SD, and TTL-CV did not differ according to sex, type of donor, retransplant, pretransplant kidney disease, body mass index, or posttransplant time; hence, they are stable in kidney transplant recipients > 5 years after KT. The higher the TTL-mean, the higher the TTL-SD. Age and the TTL-SD significantly predicted the TTL-mean (*p* < .001). Tacrolimus dose change and the TTL-mean significantly predicted the TTL-SD (*p* < .001). Tacrolimus dose change significantly predicted the TTL-CV (*p* = .008).

**Conclusion:**

In clinically stable KTRs, TTL-SD and TTL-CV change sensitively in relation to tacrolimus dose changes. Therefore, changes in TTL-SD and TTL-CV in stable KTRs with no tacrolimus dose change require medical interest and attention.

## Introduction

Kidney transplantation (KT) is a treatment that can improve the health status and quality of life of end-stage renal disease patients [1, 2]. Kidney transplant recipients (KTRs) should take immunosuppressive drugs for the rest of their lives to prevent graft rejection [3]. Tacrolimus, an immunosuppressive drug, is commonly used as a first-line regimen to prevent graft rejection after KT [4]. The tacrolimus trough level (TTL) should be measured whenever KTRs visit the hospital because the tacrolimus dose should be adjusted based on the TTL and tacrolimus has a narrow therapeutic window and toxicity [3]. Previous studies on TTL have focused on the relevance of graft rejection and TTL or the adequate TTL to prevent graft rejection [5–7]. Interestingly, the TTL can also be used as a method for monitoring adherence to tacrolimus [8]. Therefore, research is needed to understand how TTL can be used to monitor adherence to tacrolimus.

There are various ways to monitor immunosuppressive drug adherence including self-recording, counting the number of prescribed immunosuppressive drugs, electronic monitoring, and measurement of the immunosuppressive drug trough levels [9–11]. Self-recording lacks objective credibility, and counting the number of prescribed immunosuppressive drugs and electronic monitoring involve both effort and cost [8]. If a patient is taking tacrolimus as an immunosuppressive drug, measurement of the TTL is an essential test that KTRs undergo whenever they visit the outpatient department [3]. Therefore, this may be a cost-effective objective measure of adherence to tacrolimus because this does not require extra effort and cost to implement.

The TTL is affected not only by the medication itself but also by the patient’s characteristics including ethnicity, age, sex, hepatic and renal dysfunction, and genetic factors because the TTL is influenced by the absorption and metabolism of tacrolimus [4]. As the TTL is determined individually for each patient depending on the clinical course [12], the TTL can vary according by patient. Thus, it is more reasonable to monitor changes in the TTL than the TTL from one point in time for measurement of adherence to tacrolimus [4, 8]. Furthermore, the usage of mean, standard deviation, and coefficient of variation of the TTL (TTL-mean, TTL-SD, and TTL-CV, respectively) has been suggested as a means for monitoring adherence to tacrolimus [8].

If the KTRs’ condition is stable, then the TTL is generally maintained at 3–5 ng/mL for the first year after KT [4]. However, there has been little research on how TTL-mean, TTL-SD, or TTL-CV are maintained after KT. Meanwhile, after KT, there is a high probability of late rejection and graft loss as well as a gradual lowering of drug adherence over time [13, 14]. One study showed that patients in the late stages after KT (≥5 years) have lower adherence than those in the early stages after KT (≤5 years) [14]. Hence, the TTL-mean, TTL-SD, and TTL-CV in KTRs > 5 years after transplantation should be identified in order to use the TTL as a method for monitoring adherence to tacrolimus.

At least two TTL values are required to calculate the TTL-mean, TTL-SD, and TTL-CV [15]. After KT, the number of outpatient visits decreases over time, and KTRs who have passed 4 years after KT visit outpatient clinics twice a year on average [16]. (Mann et al., 2020). Thus, KTRs > 5 years after KT are expected to have measured TTL at least twice over the 2-year period.

In this study, we identified the TTL-mean, TTL-SD, and TTL-CV during a 2-year period and the factors affecting them in KTRs > 5 years after KT. The specific objectives were to investigate the TTL-mean, TTL-SD, and TTL-CV during a 2-year period in KTRs > 5 years after KT; identify the differences in the TTL-mean, TTL-SD, and TTL-CV according to general and clinical characteristics of participants (sex, type of donor, retransplant, pretransplant kidney disease, body mass index (BMI)); determine the differences amongst the TTL-mean, TTL-SD, and TTL-CV values based on tacrolimus dose change and graft rejection; evaluate the correlations amongst age, posttransplant period, TTL-mean, TTL-SD, and TTL-CV; and reveal factors affecting the TTL-mean, TTL-SD, and TTL-CV.

## Methods

### Research design

In this retrospective study, we used medical records to investigate the TTL-mean, TTL-SD, TTL-CV, and the factors affecting them over a 2-year period in KTRs > 5 years after KT.

### Participants

Participants included 248 patients amongst the 406 patients who underwent KT from 1 January 2010 to 31 December 2012, in one hospital; patients satisfied the following inclusion criteria during the 2-year period (January 1, 2017 to December 31, 2018): KTRs had undergone KT more than 5 years prior to enrolment (January 1, 2010 to December 31, 2012); were older than 18 years; maintained on tacrolimus-based triple therapy with mycophenolate mofetil and steroids; visited the outpatient department at least twice; measured the TTL at least twice. The exclusion criterion was having multiple organ transplants (e.g., liver-kidney, kidney-pancreas).

### Data collection

Data including sex, age, height, weight, transplantation day, pretransplant kidney disease, type of donor (living or deceased), retransplant, TTL, change of tacrolimus dose, and graft rejection were collected from patient medical records.

Data on age, height, weight, transplantation day, and retransplant were collected from the medical records of the last visit to an outpatient clinic in 2018. Data on TTLs measured at the outpatient clinic during routine visits from 1 January 2017 to 31 December 2018 were collected. The TTL was measured every time a patient visited for routine check-ups and immunosuppressant prescriptions; each patient had more than six TTL measurements. TTLs that were measured during hospitalization were excluded in order to obtain TTLs of medically stable patients [17]. Cases of graft rejection and tacrolimus dose change (both increase and decrease) during the same period were also investigated.

The TTL-mean, TTL-SD, and TTL-CV of the participants were calculated. The CV was calculated by dividing the SD by the mean [15]. BMI was calculated by dividing body weight by the square of height.

### Data analysis

The collected data were analyzed using SPSS for Windows version 25.0 (IBM Corp., Armonk, NY, USA). The characteristics of the participants were analyzed by descriptive statistics, and differences in the TTL-mean, TTL-SD, and TTL-CV according to the characteristics of the participants were analyzed by independent t-tests, Mann–Whitney U tests, Kruskal–Wallis tests, and analyses of variance. Correlations amongst age, posttransplant time, TTL-mean, TTL-SD, and TTL-CV were determined using Pearson’s correlation coefficient. A χ^2^-Test was used to evaluate graft rejection according to the tacrolimus dose change. Multivariable regression analysis was conducted to identify the factors influencing the TTL-mean, TTL-SD, and TTL-CV. Categorical variables were analyzed by converting them into dummy variables. A *p* value of less than .05 was considered to be significant.

## Results

### General and clinical characteristics of participants

The general and clinical characteristics of the participants are presented in Table 1. The average number of TTL measurements per patient was 11.75.

**Table 1:** Demographic and clinical characteristics of participants (*N* = 248)

Characteristics	Classification	n (%), mean ± SD (min–max)
Sex	Male	149 (60.1)
	Female	99 (39.9)
Age (years)		53.68 ± 12.18 (25–76)
Type of donor	Deceased	120 (48.4)
	Living	128 (51.6)
Retransplant	Yes	22 (8.9)
Pretransplant kidney disease	IgA nephropathy	42 (16.9)
	DM ESRD	33 (13.3)
	Glomerular nephropathy	27 (10.9)
	ADPKD	21 (8.5)
	Unknown	74 (29.8)
	HT	17 (6.9)
	Others	34 (13.7)
BMI	< 18.5	26 (10.5)
	18.5–24.9	162 (65.3)
	> 24.5	60 (24.2)
Posttransplant period (months)		90.71 ± 9.82 (75.0–110.0)
Tacrolimus dose change	Yes	93 (37.5)
Rejection	Yes	11 (4.4)
Tacrolimus trough level	Mean	6.00 ± 1.07 (2.93–9.38)
	SD	1.51 ± 1.09 (0.35–9.34)
	CV	0.25 ± 0.14 (0.70–1.20)

*Abbreviations*: *ADPKD* Autosomal dominant polycystic kidney disease, *BMI* Body mass index, *CV* Coefficient of variation, *DM ESRD* Diabetes mellitus end-stage renal disease, *HT* Hypertension, *IgA* Immunoglobulin A, *SD* Standard deviation

### Differences in TTL-mean, TTL*-*SD, and TTL*-*CV according to participant characteristics

There were no statistically significant differences amongst the TTL-mean, TTL-SD, and TTL-CV according to sex, type of donor, retransplant, pretransplant kidney disease, BMI, and graft rejection. There was no difference in the TTL-mean according to tacrolimus dose change. The TTL-SD and TTL-CV in participants with tacrolimus dose change were statistically significantly higher than those in the participants without tacrolimus dose change (both *p* < .001) (Table 2).

**Table 2:** Tacrolimus trough level according to demographic and clinical characteristics (*N* = 248)

Characteristics	Classification	Tacrolimus trough level								
		Mean	t or *F*	*p*	SD	t or *F*	*p*	CV	t or *F*	*p*
Sex	Male	6.03 ± 1.15	0.47	.638	1.44 ± 0.88		.635^†^	0.23 ± 0.11		.633^†^
	Female	5.97 ± 0.93			1.62 ± 1.35			0.26 ± 0.18		
Type of donor	Deceased	6.05 ± 1.00	0.63	.530	1.48 ± 1.15		.541^†^	0.24 ± 0.16		.167^†^
	Living	5.97 ± 1.13			1.65 ± 1.13			0.27 ± 0.15		
Retransplant	Yes	6.20 ± 1.36	0.70	.490	1.39 ± 1.20		.172^†^	0.21 ± 0.13		.085^†^
	No	5.99 ± 1.04			1.59 ± 0.13			0.26 ± 0.16		
Pretransplant kidney disease	IgA nephropathy	5.87 ± 0.90	1.24	.289	1.46 ± 0.89		.369^‡^	0.25 ± 0.14	0.70	.689^‡^
	DM ESRD	6.34 ± 1.24			1.72 ± 0.91			0.27 ± 0.13		
	Glomerular nephropathy	5.69 ± 1.16			1.38 ± 1.05			0.23 ± 0.12		
	ADPKD	6.12 ± 0.89			1.46 ± 0.93			0.23 ± 0.12		
	Unknown	5.97 ± 1.01			1.57 ± 1.38			0.25 ± 0.17		
	HT	5.93 ± 1.25			1.19 ± 0.42			0.20 ± 0.05		
	Others	6.16 ± 1.00			1.54 ± 1.18			0.25 ± 0.16		
BMI	< 18.5	5.70 ± 1.19		.073^‡^	1.39 ± 0.93		.390^‡^	0.23 ± 0.12		.153^‡^
	18.5–24.9	6.09 ± 1.03			1.48 ± 1.00			0.24 ± 0.13		
	> 24.5	5.92 ± 1.11			1.64 ± 1.37			0.27 ± 0.17		
Tacrolimus dose change	Yes	6.00 ± 1.06		.797^†^	1.76 ± 1.33		<.001^†^	0.28 ± 0.17		<.001^†^
	No	6.01 ± 1.07			1.36 ± 0.90			0.22 ± 0.12		
Rejection	Yes	5.64 ± 0.90		.129^†^	1.35 ± 0.61		.971^†^	0.23 ± 0.07		.557^†^
	No	6.02 ± 1.07			1.52 ± 1.11			0.24 ± 0.15		

^†^Mann–Whitney U test; ^‡^Kruskal–Wallis test

*Abbreviations*: *ADPKD* Autosomal dominant polycystic kidney disease; *BMI* Body mass index, *CV* Coefficient of variation, *DM ESRD* Diabetes mellitus end-stage renal disease, *HT* Hypertension, *IgA* Immunoglobulin A, *SD* Standard deviation

### Correlations amongst age; posttransplant time; and TTL-mean, TTL*-*SD, and TTL-CV

Age was positively correlated with the TTL-mean (*r* = .13, *p* = .049). The TTL-mean was positively correlated with the TTL-SD (*r* = .051, *p* < .001). Posttransplant time did not correlate with the TTL-mean, TTL-SD, and TTL-CV (Table 3).

**Table 3:** Correlations amongst age, posttransplant time, and the mean, standard deviation, and coefficient of variation of the tacrolimus trough level (N = 248)

Variables	Age	Transplantation period	TTL-mean	TTL-SD	TTL-CV
	*r* (*p*)				
Age	1	.04 (.491)	.13 (.049)	−.02 (.830)	−.06 (.348)
Posttransplant time		1	−.04 (.498)	−.04 (.548)	−.02 (.728)
TTL-mean			1	.051 (<.001)	.29 (<.001)
TTL-SD				1	.96 (<.001)

*Abbreviations*: *CV* Coefficient of variation, *SD* Standard deviation, *TTL* Tacrolimus trough level

### Graft rejection according to tacrolimus dose change

There was no graft rejection according to tacrolimus dose change (χ^2^ = 1.43, *p* = .339) (Table 4).

**Table 4:** Graft rejection according to tacrolimus dose change (*N* = 248)

Variable	Tacrolimus dose change		Total	χ^2^ *(p)*
	Yes	No		
	n (%)			
Graft rejection				
Yes	6 (54.5)	5 (45.5)	11 (4.4)	1.43 (.339)
No	87 (36.7)	150 (63.3)	237 (95.6)	
Total	93 (37.5)	155 (62.5)	248 (100)	

### Factors influencing the TTL-mean, TTL*-*SD, and TTL-CV

Multivariable regression analyses were conducted to determine factors influencing the TTL-mean, TTL-SD, and TTL-CV.

The factors influencing the TTL-mean were age (*β* = 0.13, *p* = .031) and the TTL-SD (*β* = 8.87, *p* < .001). The model had an explanatory power of 27.7%, which was significant (*F* = 27.86, *p <* .001). As a result of reviewing the basic assumptions for multivariable regression analysis, the Durbin–Watson value was 1.76, which was independent of the residuals without autocorrelation. The variance inflation factor value was 1.0, indicating no multicollinearity.

The multivariable regression model that included tacrolimus dose change and the TTL-mean significantly predicted the TTL-SD (*F* = 29.26, *p* < .001). The variance inflation factor values were 1.0, with no multicollinearity. The Durbin–Watson value was sufficient to satisfy independence. Tacrolimus dose change (*β* = − 0.18, *p* = .002) and the TTL-mean (*β* = 0.51, *p* < .001) strongly affected the TTL-SD.

Tacrolimus dose change affected the TTL-CV (*β* = − 0.21, *p* = .002). The multivariable regression model had an explanatory power of 4.4%, which was significant (*F* = 5.00, *p* = .008). The Durbin–Watson value was 1.71, which was independent of the residuals without autocorrelation, and the variance inflation factor value was 1.0 with no multicollinearity (Table 5).

**Table 5:** Factors influencing mean, standard deviation, and coefficient of variation of tacrolimus trough level (*N* = 248)

Dependent variable	Predictors	B	*β*	t	*p*	R^2^	F	*p*
TTL-mean	Constant	4.58		16.63	<.001	.277	27.86	<.001
	Age	0.01	0.13	2.17	.031			
	TTL-SD	0.50	0.52	8.87	<.001			
	Tacrolimus dose change (Yes = 0)	0.22	0.10	1.67	.097			
TTL-SD	Constant	−1.15		−2.72	.007	.287	29.26	<.001
	Age	−0.01	−0.06	−1.03	.303			
	TTL-mean	0.52	0.51	8.87	<.001			
	Tacrolimus dose change (Yes = 0)	−0.41	−0.18	−3.10	.002			
TTL-CV	Constant	0.31		7.63	.000	.044	5.00	.008
	Age	0.00	− 0.04	− 0.57	.569			
	Tacrolimus dose change (Yes = 0)	− 0.06	− 0.21	−3.10	.002			

*Abbreviations*: *CV* Coefficient of variation, *SD* Standard deviation, *TTL* Tacrolimus trough level

## Discussion

This study identified the TTL-mean, TTL-SD, and TTL-CV and evaluated the factors affecting them in 248 KTRs over 2-year period > 5 years after KT.

The TTL-mean in this study was 6.0 ng/mL. According to clinical guidelines for KT, the optimal TTL after 1 year after KT is 4–8 ng/mL [3, 18], suggesting that the participants’ TTLs met clinical guidelines for KT [3].

In this study, the TTL-mean did not depend on the general and clinical characteristics such as sex, type of donor, retransplant, pretransplant kidney disease, or BMI. Also, the TTL-mean did not differ according to posttransplant time. The TTL can be affected by various factors such as medication, the patient’s characteristics including hepatic and renal dysfunction, and clinical course [4, 12]. The TTL within 1 year after KT is not maintained at the same level because it can have a significant impact on graft function maintenance or transplant rejection [3, 7, 19]. However, if a KTR’s condition becomes stable after 1 year after KT, the TTL is generally unchanged [3]. The participants of this study were KTRs > 5 years after KT who visited outpatient clinic and were clinically stable. Also, the general characteristics may not be associated with TTL if therapeutic drug monitoring was effectively achieved [18]. Therefore, our result shows that even though the TTL is affected by the patient’s general and clinical characteristics including clinical course [4, 12], the TTL-mean in medically stable KTRs is not influenced by the general and clinical characteristics.

Regarding differences in the TTL-SD and the TTL-CV of participants, according to participants’ general and clinical characteristics, there was no differences in the study. Tacrolimus has a very narrow range of therapeutic blood levels because it increases the risk of infection if taken more than necessary and can result in transplant rejection if taken less than necessary [7]. Thus, the TTL would have been finely adjusted to maintain the therapeutic blood levels. In this study, there was no difference in the TTL-SD and TTL-CV depending on graft rejection. The average TTL-SD of participants who experience graft rejection was 1.35, which is lower than the TTL-SD cut-off score (2.5) for evaluating graft rejection [20]. Similarly, the average TTL-CV of participants who experience graft rejection was 0.23, which is lower than the TTL-CV cut-off score (0.53) for evaluating graft rejection [8]. There were not many changes in the average TTL-SD and TTL-SD over the 2-year period in the clinically stable KTRs > 5 years after KT. Accordingly, it can be surmised that there is no difference in the TTL-SD and the TTL-CV over a 2-year period according to the participants’ general and clinical characteristics if the KTRs is medically stable.

This study found that the factors affecting the TTL-mean were age and the TTL-SD. Age among the participant’s general characteristics was positively correlated with the TTL-mean, and it also influenced the TTL-mean but not the TTL-SD or the TTL-CV. The TTL-mean could be higher in old recipients than in young recipients even though they take the same dose of tacrolimus; because old recipients have lower tacrolimus clearance rates than young recipients [21, 22]. As such, age may affect the TTL-mean but not to the extent that it affects the TTL-SD and the TTL-CV.

The TTL-SD influenced the TTL-mean; the greater the TTL-SD, the higher the TTL-mean. Patients with lower immunosuppressant adherence may have a higher mean and SD immunosuppressant trough level because they take more than the prescribed dose before undergoing blood test for TTL measurement to make up for missing doses [8]. However, it is difficult to conclude in this study that the TTL-SD affects the TTL-mean because of nonadherence to tacrolimus as the participants’ adherence to tacrolimus was not measured in this study. Therefore, further research is necessary to identify the relationships amongst the TTL-mean, TTL-SD, and adherence to tacrolimus.

Factors affecting the TTL-SD were the TTL-mean and tacrolimus dose change. The TTL-mean was the main factor affecting the TTL-SD; the higher the TTL-mean, the greater the TTL-SD. This characteristic may appear in KTRs with low adherence to tacrolimus [8], as noted above, or may have other causes, which may require further research.

As tacrolimus dose change affects the TTL [23], tacrolimus dose change would influence the TTL-SD. In this study, 37.5% of the patients underwent tacrolimus dose changes. If the TTL is not maintained within the therapeutic range or if health problems such as infection or cancer occur, the immunosuppressant dose may be changed temporarily or permanently [3, 23]. In this study, not all tacrolimus dose change reasons were recorded in the medical records, therefore, the reasons for the tacrolimus dose changes were not investigated. However, it could be supposed that the reasons for the tacrolimus dose changes were not serious health problems as the participants were outpatients assumed to be medically stable and not recording may also indicate that the reasons were not serious. Tacrolimus is a highly toxic drug [24]; thus, if the patient is medically stable, the tacrolimus dose change may have been limited within the therapeutic blood range. Nevertheless, the tacrolimus dose change affected the TTL-SD suggesting that the TTL-SD is an indicator that responds sensitively to change in the dosage of tacrolimus. The only factor affecting the TTL-CV was the tacrolimus dose change. As the TTL-CV is calculated using the TTL-mean and TTL-SD [15], tacrolimus dose change, which affected the TTL-SD, would influence the TTL-CV. As the participants were clinically stable KTRs, there were not many changes in TTL-CV. The TTL-CV is also an indicator that responds sensitively to tacrolimus dose change.

Therefore, if there is change in TTL-SD and TTL-CV in KTRs with no change in tacrolimus dose and medical stability, it is necessary to identify what causes the change in TTL-SD and TTL-CV. It is predicted that various factors such as non-adherence, time of tacrolimus administration, and interaction of food and other drugs might have an effect [4, 8, 25], and active intervention will be required if non-adherence is the cause. In addition, studies have been shown that indicators related to tacrolimus trough level such as intra-patient tacrolimus level variability and TTL-CV are associated with graft survival or non-adherence [8, 25]. Development of a computer program in which variables figures such as TTL-mean, TTL-SD, and TTL-CV are calculated automatically using tacrolimus trough level will help health professionals easily identify and evaluate TTL-mean, TTL-SD, and TTL-CV of KTRs.

This study has a few limitations. First, the participants were KTRs from a single university hospital. Ethnicity can influence TTL [4], however, all of participants are Korean. There is a possibility of selection bias because the study targeted patients who maintained kidney function for more than 5 years. Therefore, generalization of the study’s finding is difficult, and a future study using a representative sample should be conducted. Second, the study did not identify the association of the TTL-mean, TTL-SD, and TTL-CV with adherence to tacrolimus. Further studies are needed to evaluate this association and to determine whether the TTL-mean, TTL-SD, and TTL-CV can be used as variables to measure adherence to tacrolimus. Third, the effects of dose and change of dose of mycophenolate mofetil and steroid on graft rejection were not considered. In the future, studies should be conducted considering the effects of tacrolimus, and other immunosuppressive medications on graft rejection. Nevertheless, our findings identified the TTL-mean, TTL-SD, and TTL-CV, and the factors affecting them over a 2-year period in KTRs > 5 years after KT. Our results could be used to develop methods to monitor adherence to tacrolimus using the TTL-mean, TTL-SD, and TTL-CV in KTRs.

## Conclusions

This study identified the TTL-mean, TTL-SD, and TTL-CV and the factors affecting them over a 2-year period in KTRs > 5 years after KT. The TTL-mean, TTL-SD, and TTL-CV did not differ according to sex, type of donor, retransplant, pretransplant kidney disease, BMI, or posttransplant time; hence, they are stable in KTRs > 5 years after KT. The higher the TTL-mean, the higher the TTL-SD. Age and the TTL-SD affected the TTL-mean. Tacrolimus dose change predicted TTL-SD and TTL-CV. The TTL-SD and TTL-CV are sensitive to tacrolimus dose change. Thus, if there is change in TTL-SD and TTL-CV in KTRs with no change in tacrolimus dose and medical stability, the causes must be identified. Health professionals need monitor to whether KTRs are taking tacrolimus properly if there are KTRs with high TTL-mean and TTL-SD. Future studies are necessary to identify the association of the TTL-mean, TTL-SD, TTL-SV and adherence to tacrolimus.

## Data Availability

The de-identified datasets generated and/or analysed during the current study are available from the corresponding author on reasonable request**.**

## Abbreviations

TTL: Tacrolimus trough level
TKR: Kidney transplant recipients
SD: Standard deviation
CV: Coefficient of variation

## References

[1] Jeon KO, Son SY, Hahm MI, Kim SI (2015). Quality of life among end-stage renal disease treatments and economic evaluation of renal transplantation and hemodialysis treatments. J Korean Soc Transplant.

[2] Yoo HJ, Kim KS (2013). Relationship between stress and the quality of life among the recipients of the living donor liver transplantation. J Korean Clinic Nurs Res.

[3] Cho WH, Kim HT, Park WY (2017). Renal transplantation.

[4] Yu M, Liu M, Zhang W, Ming Y (2018). Pharmacokinetics, pharmacodynamics and pharmacogenetics of tacrolimus in kidney transplantation. Curr Drug Metab.

[5] Huang C-T, Shu K-H, Ho H-C, Wu M-J (2016). Higher variability of tacrolimus trough level increases risk of acute rejection in kidney transplant recipients. Transplant Proc.

[6] Jung H-Y, Cho S-Y, Choi J-Y, Cho J-H, Park S-H, Kim Y-L (2019). Comparison of transplant outcomes for low-level and standard-level tacrolimus at different time points after kidney transplantation. J Korean Med Sci.

[7] Yin S, Song T, Jiang Y, Li X, Fan Y, Lin T (2019). Tacrolimus trough level at the first month may predict renal transplantation outcomes among living Chinese kidney transplant patients: a propensity score-matched analysis. Ther Drug Monit.

[8] Hsiau M, Fernandez HE, Gjertson D, Ettenger RB, Tsai EW (2011). Monitoring nonadherence and acute rejection with variation in blood immunosuppressant levels in pediatric renal transplantation. Transplantation..

[9] Jung H-Y, Seong SJ, Choi J-Y, Cho J-H, Park S-H, Kim C-D (2017). The efficacy and stability of an information and communication technology-based centralized monitoring system of adherence to immunosuppressive medication in kidney transplant recipients: study protocol for a randomized controlled trial. Trials..

[10] Marsicano EO, Fernandes NS, Colugnati FA, Fernandes NMS, De Geest S, Sanders-Pinheiro H (2015). Multilevel correlates of non-adherence in kidney transplant patients benefitting from full cost coverage for immunosuppressives: a cross-sectional study. PLoS One.

[11] Hwang YH, Park SJ (2020). Compliance in kidney transplant recipient including compliance with immunosuppressive medication. [a concept analysis of compliance in kidney transplant recipient including compliance with immunosuppressive medication]. J Korean Biol Nurs Sci.

[12] Scheel J, Reber S, Stoessel L, Waldmann E, Jank S, Eckardt K-U (2017). Patient-reported non-adherence and immunosuppressant trough levels are associated with rejection after renal transplantation. BMC Nephrol.

[13] Pollock-Barziv SM, Finkelstein Y, Manlhiot C, Dipchand AI, Hebert D, Ng VL (2010). Variability in tacrolimus blood levels increases the risk of late rejection and graft loss after solid organ transplantation in older children. Pediatr Transplant.

[14] Lee JA, Kim YA, Cho Chung HI (2019). Factors affecting treatment adherence of kidney transplantation recipients. J Korea Contents Assoc.

[15] Hima BK, Morusupalli R, Dey N, Rao CR (2019). Coefficient of variation and machine learning applications.

[16] Mann S, Naylor KL, McArthur E, Kim SJ, Knoll G, Zaltzman J (2020). Projecting the number of Posttransplant clinic visits with a rise in the number of kidney transplants: a case study from Ontario, Canada. Can J Kidney Health Dis.

[17] Shemesh E, Shneider BL, Savitzky JK, Arnott L, Gondolesi GE, Krieger NR, Kerkar N (2004). Medication adherence in pediatric and adolescent liver transplant recipients. Pediatrics..

[18] Gaynor JJ, Ciancio G, Guerra G, Sageshima J, Roth D, Chen L (2014). Predictors of reduced tacrolimus dose and trough level through 36 months post-transplant among 578 adult primary kidney transplant recipients. Clin Transpl.

[19] Žilinská Z, Dedinská I, Breza J, Laca L (2016). Impact of trough levels of tacrolimus on kidney function and graft survival in short and longer periods after renal transplantation. Transplant Proc.

[20] Stuber ML, Shemesh E, Seacord D, Washington J, Hellemann G, McDiarmid S (2008). Evaluating non-adherence to immunosuppressant medications in pediatric liver transplant recipients. Pediatr Transplant.

[21] David-Neto E, Romano P, Kamada Triboni AH, Ramos F, Agena F, Rezende Ebner PA (2017). Longitudinal pharmacokinetics of tacrolimus in elderly compared with younger recipients in the first 6 months after renal transplantation. Transplantation..

[22] Krenzien F, Quante M, Heinbokel T, Seyda M, Minami K, Uehara H (2017). Age-dependent metabolic and immunosuppressive effects of tacrolimus. Am J Transplant.

[23] Lancia P, Jacqz-Aigrain E, Zhao W (2015). Choosing the right dose of tacrolimus. Arch Dis Child.

[24] Sikma MA, van Maarseveen EM, van de Graaf EA, Kirkels JH, Verhaar MC, Donker DW (2015). Pharmacokinetics and toxicity of Tacrolimus early after heart and lung transplantation. Am J Transplant.

[25] Süsal C, Döhler B (2019). Late intra-patient tacrolimus trough level variability as a major problem in kidney transplantation: a collaborative transplant study report. Am J Transplant.

